# Physicochemical, microbiological, and sensory characteristics of “*Sui Wu’u*” traditional pork products from Bajawa, West Flores, Indonesia

**DOI:** 10.14202/vetworld.2023.1165-1175

**Published:** 2023-05-31

**Authors:** Yuliana Tandi Rubak, Herianus J. D. Lalel, Maxs Urias Ebenhaizar Sanam

**Affiliations:** 1 Department of Agrotechnology, Faculty of Agriculture, Universitas Nusa Cendana, Kupang, East Nusa Tenggara 85228, Indonesia; 2 Department of Animal Diseases Sciences and Veterinary Public Health, Faculty of Veterinary Medicine, Universitas Nusa Cendana, Kupang, East Nusa Tenggara 85228, Indonesia

**Keywords:** Bajawa, meat preservation, sensory characteristics, *Sui Wu’u*

## Abstract

**Background and Aim:**

*Sui Wu’u* is a traditional meat preservation product from Bajawa, a region in East Nusa Tenggara. It is made by mixing pork with salt and corn flour, which is then stored in a bamboo container (*tuku)* for months. After 6 months of storage, this study examined the physicochemical, microbiological, and sensory properties of *Sui Wu’u*.

**Materials and Methods:**

*Sui Wu’u* products were prepared using the traditional recipe from the Bajawa community. Fresh pork (pork belly and backfat), corn flour, and salt were purchased from local/traditional markets at proportions of 65%, 30%, and 5%, respectively. The physicochemical, amino acid, fatty acid profile, microbiological, and sensory properties of *Sui Wu’u* were evaluated after being stored for 6 months in a bamboo container (*tuku*).

**Results:**

The results indicated that these *Sui Wu’u* were mainly characterized by high-fat levels, followed by protein. The pH value, salt content, moisture content, and water activity were 4.72%, 1.72%, 6.11%, and 0.62%, respectively. Minerals (K, P, Se, and Zn) and vitamin B6, as well as amino acids, such as leucine, phenylalanine, lysine (essential amino acids), glycine, proline, glutamic acid, and alanine (non-essential amino acids), are present in *Sui Wu’u*. The fatty acid profile was dominated by monounsaturated fatty acids (MUFA) (21.69%), saturated fatty acids (SFA) (17.78%), and polyunsaturated fatty acids (PUFA) (5.36%). Monounsaturated fatty acids, oleic acid (C18:1n9) was the most abundant fatty acid in *Sui Wu’u*, followed by palmitic acid SFA (C16:0); MUFA stearic acid (C18:0); and PUFA linoleic (C18:2n-6). The microbiological characteristics of *Sui Wu’u* showed no detectable microorganisms (<10 CFU/g) for *Salmonella*, total *E. coli* and total *Staphylococcus*, and average values of 4.4 × 10^5^ CFU/g for total microbes, which were still below the maximum limit of microbial contamination according to the regulations of the Food and Drug Supervisory Agency of the Republic of Indonesia. The sensory assessment indicated that panelists highly preferred (rated as very like) *Sui Wu’u* for all sensory attributes.

**Conclusion:**

The physicochemical, microbiological, and sensory characteristics of *Sui Wu’u* after 6 months of storage indicated that it still provides essential nutrients for the body and is quite safe for consumption. The stability of *Sui Wu’u’s* shelf life can be attributed to the appropriate combination of pork, salt, corn flour, bamboo packaging (*tuku*), and storage temperature. The high-fat content in *Sui Wu’u* can be reduced by increasing the proportion of lean meat. Ensuring strict sanitation during the manufacturing process, using high-quality pork, salt, corn flour, and proper packaging with bamboo can further improve the safety of *Sui Wu’u* for consumption.

## Introduction

Food preservation has been a well-known practice since ancient times and is often regarded as a form of local wisdom in a particular area. The purposes and methods of food preservation vary widely depending on culture, the abundance of particular foods in a region, and climatic conditions [1, 2]. *Sui Wu’u* is a cured pork product from Bajawa, East Nusa Tenggara, Indonesia (*Sui* means pork; *Wu’u* means corn flour). The origin of *Sui Wu’u* is associated with the *Reba* traditional ceremony in the Bajawa community, which involves slaughtering a pig as part of the ceremony. Leftover pork and pork back fat that have not been consumed during the ceremony, will be transformed into cured products by mixing salt and corn flour and then placed in bamboo containers (*tuku*). The *tuku* filled with meat is stored at ambient temperature for months to several years. During ripening, food components such as proteins, fats, and carbohydrates break down into simpler components partly due to the microbial activity [3]. The changes in these components can be both beneficial and unfavorable. The advantages include increased digestibility and bioavailability of the final product [4] and the formation of various bioactive components [5], which have antioxidant, cholesterol-lowering, angiotensin-converting enzyme inhibition, anti-diabetic, anti-aging, anticancer, immunomodulatory, and antimicrobial properties [6, 7]. On the other hand, the disadvantage is the formation of undesired metabolites, such as biogenic amines and toxins, produced by pathogenic microbes during meat spoilage [8, 9].

Studies have revealed that preservation can improve the sensory qualities and maintain the microbiological and physicochemical qualities of the final product. However, the success of food preservation is highly dependent on several factors, including raw materials, preservatives, methods of preservation, packaging, temperature, and storage duration [10, 11]. A suitable combination of these factors will determine the shelf life and quality of the preserved food [12]. Salt is the most commonly used additive for the traditional preservation of meat and fish products [13, 14]. Salt contributes to the formation of flavor and plays a role in the formation of bioactive components [15]. The most important function of salt is to extend the shelf life of products, as it can inhibit the growth of spoilage bacteria [16]. Furthermore, salt concentration is crucial in determining the types of microbes that can thrive in the preserved product. Peda, Jambal Roti, Inasua, Urutan Bali, Tukai, and Fish Sauce are examples of some traditional Indonesian food products preserved with salt [17, 18]; others are Doenjang and Jeotgal (Korea) [19, 20], Pla-ra (Thailand) [21], and Prohok (Cambodia) [22]. Meat preservation is often achieved by combining salt with carbohydrate sources such as rice, coconut sugar, spices, and other ingredients [18, 21]. Each ingredient used contributes to the shelf life of the cured meat. Adding carbohydrate sources reduces moisture and provides energy for microbial growth during storage. Interestingly, *Sui Wu’u* products use corn flour as an additional ingredient, which is not found in traditional meat-based products. Obtaining comprehensive information on the physicochemical, microbiological, and sensory properties of a traditional food product is crucial for future development and improvement.

This study aimed to investigate the physicochemical, microbiological, and organoleptic properties of *Sui Wu’u*, a traditional pork product from Bajawa, East Nusa Tenggara, after a 6-month storage period.

## Materials and Methods

### Ethical approval

No ethical approval was required for this study. However, samples were collected as per standard sample collection methods.

### Study period and location

The study was conducted from March 2022 to December 2022. *Sui Wu’u* samples were made and stored in Bajawa, West Flores. Indonesia for six months. The average temperature during storage of the *Sui Wu’u* sample is the daily maximum temperature of 24°C and minimum temperature of 16°C. Physicochemical analysis, microbiology, and sensory properties were investigated at Nusa Cendana University, IPB University, and Saraswanti Indo Genetech Laboratory (SIG), Bogor, Indonesia.

### Preparation of the *Sui Wu’u* sample

The *Sui Wu’u* sample was prepared following a recipe from the Bajawa community that has been passed down for generations. To produce *Sui Wu’u*, pork belly and backfat were used along with salt and corn flour at 65%, 30%, and 5%, respectively. All ingredients were purchased from local/traditional markets. Initially, unwashed pork was cut into medium-sized pieces, sprinkled with salt, stirred until evenly distributed, and then allowed to stand for 5 min. The meat was then coated with corn flour until thoroughly covered and stuffed into the *tuku*, alternating layers of meat with the remaining corn flour. Afterwards, the *tuku* was closed tightly and stored in the kitchen area for a predetermined amount of time. After 6 months of storage, the samples were collected and transported to the laboratory under refrigeration (<4°C). The samples were then analyzed for the physicochemical (proximate, mineral, vitamins, amino acids, and fatty acid profiles), microbiological (total plate count, total *Salmonella*, *Escherichia coli*, and *Staphylococcus aureus*), and sensory properties (hedonic test).

### Physicochemical analysis

The proximate analysis includes protein, fat, moisture content, and ash [23]. All chemical components (except moisture) are expressed as a percentage of dry matter. The water activity was measured using a water activity meter (Rotronic, US). To determine the pH value, a solution of 10 g of *Sui Wu’u* was dissolved in 70 mL of distilled water and then subjected to a pH meter (pH = 700 Eutech) that was calibrated at pH = 4.0 and 7.0. The salt content was determined by the titrimetric method and the mineral content was determined by a flame atomic absorption spectrometer and a flame photometer instrument (Shimadzu AA 6300 AAS, Japan).

### Amino acid determination

The amino acid composition of *Sui Wu’u* was analyzed using liquid chromatography with tandem mass spectrometry (LC/MS-MS) liquid chromatography system (Shimadzu). Approximately 0.5 g of sample was weighed into a 20 mL headspace glass vial with a crimp cap, then stored in a cooling water bath, and placed in the freezer at −20°C for at least 15 min. After storage, 480 μL of the oxidation agent was added. The oxidation proceeded at room temperature without a cap for 1 h in a fume hood with gentle shaking, and the sample was stored in the freezer (−20°C) for 16 h. Afterward, the sample was thawed, and 60 mg of sodium bisulfite was added. The samples were left in a fume hood for ≥3 h to let sulfur dioxide gas escape. After all, the gas had evaporated, 3 mL of hydrochloric acid (6 M with 0.1% w/v phenol) was added, then the vial was sealed and placed in a preheated oven at 110°C for 21 h. After hydrolysis, the samples were allowed to cool to the handling temperature and then neutralized with 4 mL sodium hydroxide (6 M), mixed thoroughly, and left to cool until the handling temperature. After cooling, the sample was transferred to a falcon tube and filled to a final volume of 25 mL with distilled water. The sample was then set to pH = 2.20, transferred into a 2 mL tube, and centrifuged. An aliquot was filtered through a 0.2 mm and injected into LC-MS/MS system.

### Fatty acid profile

The capillary gas chromatography method was used to determine the fatty acid composition. Samples and fatty acid methyl esters (FAME) were derived and extracted according to the method described by Ratnayake *et al*. [24]. The extraction process was performed using the Soxhlet method. Subsequently, 20 g of oil-form fat was weighed and subjected to further analysis through the methylation process to form fatty acid derivative compounds, methyl esters. The methylation process was performed by refluxing fatty acids in a water bath using NaOH-methanol, isooctane, and BF_3_ solvents. A total of 20 mg of sample was put into a test tube, and 1 mL of 0.5 N NaOH-methanol was added, followed by heating for 20 min and then cooling. Two milliliters of BF_3_ solution (20%) and 5 mg/mL of the internal standard were added, followed by heating for 20 min and then cooling. The mixture was shaken carefully after adding 2 mL of saturated NaCl and 1 mL of isooctane. The formed isooctane layer was transferred using a pipette into a tube containing 0.1 g of anhydrous Na_2_SO_4_, and then allowed to stand for 15 min. The liquid fraction formed was then separated, while the oil fraction formed (1 μL) was injected into gas chromatography. Separation and quantification of FAME were performed using a gas chromatograph (Shimadzu) equipped with a Supelco SP™-2560 (Sigma-Aldrich, USA) fused silica capillary column (internal diameter 100 m × 0.25 mm × film thickness 0.20 m). Nitrogen was used as the carrier gas, and the oven temperature was initially maintained at 225°C, then raised to a final temperature of 240°C (2°C/min). The injector and detector temperatures were maintained at 250°C.

### Sensory evaluation

A total of 30 semi-trained panelists, including food technology lecturers and students from the Agrotechnology Department, Faculty of Agriculture, Nusa Cendana University, were involved in conducting a sensory evaluation of *Sui Wu’u* products. Sensory evaluation was conducted between 11.00 and 12.00 WIB, according to the procedure by Chambers and Wolf [25] with some modifications. A sample of grilled *Sui Wu’u* (30 g) was received by the panelists to be evaluated for color, aroma, juiciness, softness, and overall acceptability. A five-point hedonic scale (“extremely dislike,” “dislike,” “slightly dislike,” “like,” and “extremely like”) was employed for evaluation. For comparison, panelists also received grilled pork that had not undergone the storage process.

### Microbiological analysis

A sample of *Sui Wu’u* (25 g) was collected aseptically and placed in a sterile tube containing 45 mL of 0.1% sterile *Butterfield’s phosphate-buffered dilution water* (Merck). Serial decimal dilutions were prepared in sterile 0.1% (wt/vol) peptone water for microbiological testing. Total aerobic mesophilic microflora was enumerated in Potato Count Agar (Oxoid, UK) after 48 h of incubation at 35°C according to Maturin and Peeler [26]; total *E. coli* in Eosin Methylene Blue Agar (Oxoid) after 48 h of incubation at 35°C according to Nuraida *et al*. [27]; total *Salmonella* in Xylose Lysine Deoxycholate Agar (Oxoid) after 48 h of incubation at 35°C according to Brichta-Harhay *et al*. [28]; and total *S. aureus* according to ISO 6888-1:1999 Amd 2 [29] using Baird-Parker agar (BPA) specific media (Oxoid) plus egg yolk with the spread plate method after incubation at 37°C for 48 h. Typical *S. aureus* colonies on BPA media are round and black with a clear zone around the colony. After incubation, plates with 30–300 colonies were counted. Microbiological data are presented in colony-forming units (CFU)/g.

### Statistical analysis

Proximate analysis was carried out in triplicate (n = 3) for each sample, except for amino acid and fatty acid analysis. Data are presented as the mean ± standard deviation. All statistical analyses were performed using the statistical package for the social sciences version 16 (IBM Corp. USA).

## Results and Discussion

### Physicochemical analysis

The chemical composition of meat can undergo changes during prolonged storage. The magnitude of this change depends on the storage period, raw materials, and processing [30, 31]. According to the physicochemical properties (Table-1), fat was the highest component in *Sui Wu’u* (59.68%). Fat is an essential source of energy for the body, the primary energy component of meat products, and a carrier component of fat-soluble vitamins (A, D, E, K). Fat positively affects the sensory properties of meat products, especially freshness, juiciness, and lipolysis [32]. The main raw materials of *Sui Wu’u* products are pork belly and backfat which contribute to increasing its fat content. Backfat is a piece of meat taken from the back of a pig. However, the abdomen is generally classified as a cut of crude fat and, in contrast to other simple carcass cuts, has a higher fat content than the amount of muscle [33]. Pork sausage is a product that uses 20%–80% backfat and has a fat content ranging from 14% to 65% [34, 35].

**Table-1:** Physicochemical properties of *Sui Wu’u* after 6 months of storage.

Component	Concentration (mean ± SD)
Moisture (%)	6.11 ± 0.09
Ash (%)	2.76 ± 0.05
Total Fat (%)	59.68 ± 0.07
Proteins (%)	24.29 ± 0.21
Vitamin B6 (mg/100 g)	0.06 ± 0.00
Potassium (mg/100 g)	257.82 ± 0.63
Phosphorus (mg/100 g)	104.25 ± 0.93
Selenium (mcg/100 g)	11.44 ± 0.01
Zinc (mg/100 g)	1.65 ± 0.01
Salt content (%)	1.79 ± 0.03
Water activity (a_w_)	0.71 ± 0.00
pH	4.72 ± 0.00

The pH value of the *Sui Wu’u* sample was 4.27. This value indicates a decrease in pH, where the pH of fresh pork is in the range of 5.4–5.9 [36]. The pH value obtained was lower than that of *alheira* sausage in the study by Teixeira *et al*. [37], with a percentage of pork backfat and pork belly of 30%, similar to the study by Lee *et al*. [33]. The decrease in the pH of food during prolonged storage can be attributed to various factors, such as the activity of microbes, including lactic acid bacteria [18], the composition of raw materials used, and the process itself [33, 38].

The water activity of the *Sui Wu’u* sample was 0.71, which was lesser than that of traditional pork sausage [33, 39]. The low a_w_ value is a crucial factor in determining the shelf life of food products. These results indicate that *Sui Wu’u* could be stored for months. The combination of corn flour and salt in making *Sui Wu’u* is crucial in absorbing the moisture content of the meat, which results in low water content and the a_w_ value of the sample.

Traditional meat products are generally considered healthy due to their natural ingredients. However, these materials must be used in balanced proportions, like salt. The salt content of the *Sui Wu’u* sample was measured at 1.79%, while salt added in meat products generally ranges from 0.73% to 9% [40]. The percentage depends on the purpose, storage time, and combination with other ingredients. Salt plays various roles in processed meat products, such as enhancing the flavor and taste, influencing the processing stability and cooking results, and improving the water-holding capacity of meat products [41]. High salt content increases the shelf life of meat as it can inhibit the growth of spoilage microorganisms [42]. However, the high salt content in meat products has been associated with adverse health effects. Several studies have been conducted to reduce the use of salt in food products [43].

The ash content of the *Sui Wu’u* sample showed a value of 2.76%. Similar results were reported by Teixeira *et al*. [37] on *alheiras* pork sausage (2.1%–2.9%). Likewise, lamb and goat sausages with 30% pork backfat have ash content values of 3.70% and 3.75%, respectively [39]. Pork and its processed products are also good sources of minerals and vitamins [44, 45]. *Sui Wu’u* contained varying amounts of minerals K, P, Se, and Zn. Potassium is crucial for muscle contraction, nerve conduction, and providing acid-alkaline balance in the body. In contrast, mineral P is essential for energy conversion and membrane transport in the body. The minerals K and P in *Sui Wu’u* were 257.82 mg/100 g and 104.25 mg/100 g, respectively. Tomovic *et al*. [46] reported that the mineral contents of K and P (mg/100 g) in raw pork were 400 and 223, respectively. The body requires limited quantities of trace elements, including Se and Zn, as they play a key role in metabolic processes and serve as defense mechanisms against oxidative stress. The recommended dietary allowance (RDA) for these elements was 55 mg for Se, 11 mg (women), and 8 mg (men) for Zn. The concentrations of minerals Se and Zn in the samples were 11.44 mcg/100 g and 1.65 mg/100 g, respectively. Hu *et al*. [47] reported levels of Zn and P in pork samples in the range of 10.25–51.44 mg/kg and 1.68–1.97 g/kg, respectively. *Sui Wu’u* also contained vitamin B6, which is involved in immune and inflammatory responses. The RDA for vitamin B6 will vary by age, with average requirements of 1.3–1.7 mg/day.

The protein content of *Sui Wu’u* was 24.29%, which was higher than the protein content in pork sausage with 100% pork backfat as raw material [48], and traditional sausage with protein content in the range of 4.8–55.5 g/100 g [34]. The amino acid profile analysis results showed the presence of essential and non-essential amino acids (Table-2). The amino acid profile is an important index for evaluating the nutritional value of pork. Amino acids are a component of taste precursors. Hence, the amino acid content in meat will affect its sensory properties [49]. Eighteen types of amino acids were detected in *Sui Wu’u*, consisting of eight essential amino acids and ten non-essential amino acids (Table-2). Essential amino acids with the highest concentrations were leucine, phenylalanine, and lysine. The non-essential amino acids are glycine, proline, glutamic acid, and alanine. Pork also contains several amino acids like other meats [49]. The amino acid profile can be influenced by the breed of the pig [50].

**Table-2:** Amino acid composition of *Sui Wu’u* after 6 months of storage.

Amino acid	Concentration (mg/g sample)
L-Serine	9.15 ± 0.03
L-Glutamic acid	16.96 ± 0.06
L-Phenylalanine	7.86 ± 0.02
L-Isoleucine	4.15 ± 0.01
L-Valine	6.60 ± 0.03
L-Alanine	14.01 ± 0.06
L-Arginine	19.70 ± 0.08
Glycine	43.16 ± 0.19
L-Lysine	6.74 ± 0.03
L-Aspartic acid	9.34 ± 0.03
L-Leucine	9.02 ± 0.03
L-Tyrosine	3.67 ± 0.01
L-Proline	21.52 ± 0.09
L-Threonine	5.94 ± 0.02
L-Histidine	3.51 ± 0.01
L-Tryptophan	0.30 ± 0.00
L-Cystine	1.18 ± 0.01
L-Methionine	0.06 ± 0.00

### Fatty acid profile

Fatty acid composition is an essential index of meat quality. The storage of meat for a certain period can cause a decrease in the nutritional quality of the product with an increase in saturated fatty acids (SFA) and a decrease in unsaturated fatty acids (UFA). Saturated fatty acids are associated with the emergence of diseases in the circulatory system [51]. In contrast, monounsaturated fatty acids (MUFA) contribute to the cardiovascular system; whereas, polyunsaturated fatty acids (PUFA) have anti-atherosclerosis, anti-inflammatory, and anti-aggregation properties [52]. The fatty acid profile of pork is determined mainly by the breed [53], sex [54], and variations in feeding [55]. The fatty acid profile of *Sui Wu’u* is presented in Table-3.

**Table-3:** Fatty acid composition of *Sui Wu’u* after 6 months of storage (mean ± standard error).

Fatty acid	Concentration (%)
C 10:0	0.02 ± 0.00
C 12:0	0.05 ± 0.00
C 13:0	0.01 ± 0.00
C 14:0	0.54 ± 0.11
C 15:0	0.01 ± 0.00
C 16:0	10.61 ± 0.21
C 17:0	0.08 ± 0.00
C 18:0	6.27 ± 0.11
C 20:0	0.17 ± 0.00
C 22:0	0.02 ± 0.00
C 16:1	0.70 ± 0.01
C 17:1	0.07 ± 0.00
C 18:1n-9	20.33 ± 0.39
C 20:1	0.57 ± 0.00
C 22:1	0.01 ± 0.00
C 18:2n-6	4.72 ± 0.08
C 20:2n-6	0.33 ± 0.00
C 18:3n-3	0.16 ± 0.00
C 20:3n-6	0.04 ± 0.00
C 20:3n-3	0.05 ± 0.00
C 20:4n-6	0.07 ± 0.00
∑SFΑ	17.78 ± 0.34
∑MUFΑ	21.69 ± 0.42
∑PUFA	5.36 ± 0.10
PUFA/SFA	0.30 ± 0.00
PUFA-n-3	0.22 ± 0.00
PUFA-n-6	5.15 ± 0.10
PUFA-n-6/n-3	23.93 ± 0.00
AI	0.47 ± 0.00
TI	1.24 ± 0.00

SFA=Saturated fatty acids, MUFA=Monounsaturated fatty acids, PUFA=Polyunsaturated fatty acids, AI=Atherogenic index, TI=Thrombogenic index

Twenty fatty acids were found in *Sui Wu’u*, with MUFA (21.69%) as the highest concentration, followed by SFA (17.78%) and PUFA (5.36%). The value of SFA obtained is lower than that of Frankfurter pork sausage (SFA: 33.14 ± 0.64) [56], and is consistent with other traditional pork sausages, which are also dominated by lard [57]. The percentage of fatty acids in meat highly depends on the raw material. *Sui wu’u* is primarily composed of pork backfat. Vargas-Ramella *et al*. [48] showed that backfat pork was dominated by MUFA 51.72%, SFA 39.60%, and PUFA 8.68%. The percentage of SFA in meat products is 38%–43%, while the UFA (especially PUFA) and PUFA/UFA index values are low. However, MUFA is relatively high, accounting for 39%–52% [58]. Monounsaturated fatty acids, oleic acid (C18:1n9), are the most abundant fatty acids present in *Sui Wu’u*, followed by palmitic acid SFA (C16:0); MUFA stearic acid (C18:0); and PUFA linoleic acid (C18:2n-6). These results agree with reports on the fatty acid composition of products formulated with lard [37]. The fatty acids C18:1n9 and C18.2n-6 are the dominant fatty acids in pork backfat [59]. The PUFA/SFA ratio is generally used as an indicator to assess the nutritional quality of food. The sample PUFA/SFA ratio was 0.30, lower than that of traditional *alheira* sausage products [37], and slightly below the PUFA/SFA ratio suggested by World Health Organization/Food and Agriculture Organization experts, which should be above 0.4 [60]. The diets PUFA/SFA ratio of 1.0–1.5 is in a good range, which helps to reduce the risk of cardiovascular disease [61].

Polyunsaturated fatty acids (n-3) are essential constituents of cell membranes that play multiple functions through regulating membrane fluidity, eicosanoid synthesis, cell signaling, and gene expression. They contribute to the cardiovascular system and reduce the incidence of atherosclerosis, cancer, and hyperlipidemia [62]. The n-3 PUFA has a strong anti-inflammatory effect, while the n-6 PUFAs tend to be pro-inflammatory. The value of n-3 PUFA obtained in this study was lower than that of n-6 PUFA, resulting in an n-6/n-3 ratio of 23.93% (Table-3). Several studies [37, 63] have reported that pork-based products, such as pork sausage, had n-3 PUFA values in the range of 0%–2%, with an n-6/n-3 PUFA ratio ranging from 10% to 20%.

A high intake of n-3 PUFA and a balanced n-6/n-3 ratio in the body is essential for human health. The n-6/n-3 PUFA ratio has been used as a new index to replace the PUFA/SFA ratio to determine the physiological effects exerted on the body by n-6 and n-3 intake [64]. The n-6/n-3 ratio helps measure the PUFA balance in food. Some diets’ n-6/n-3 ratios are 4:1 and 7.5/1 [65]. At the same time, the contemporary Western diet is characterized by a ratio of about 10–25/1. However, the current general recommendation is that the intake of n-3 PUFAs should be higher than that of n-6. The study by Ma *et al*. [66] showed that a low ratio of n-6/n-3 PUFA (1:1) considerably reduced serum levels of tumor necrosis factor, interleukin-1, and interleukin-6 and monocyte chemoattractant protein-1, compared to the control (ratio n-6/n-3:36/1). A low ratio of n-6/n-3 PUFA enhances the inflammatory response and increases the risk of obesity [67, 68]. This is similar to the findings of a meta-analysis study by Nindrea *et al*. [69]. The low dietary intake of n-6/n-3 PUFAs was associated with a lower risk of breast cancer in Asian countries than in Western countries. However, in a review of PUFA by Davinelli *et al*. [70], it was explained that, at present, there is no consensus on which index best reflects PUFA status in the body. The role of n-6/n-3 PUFA ratio as a biomarker of disease risk or an essential index associated with inflammatory pathways is still debated. Current clinical evidence is insufficient to demonstrate that a low dietary intake of n-6 PUFA is associated with a reduced risk of coronary heart disease, and there is no correlation between a high intake of n-6 and inflammation [70]. The accuracy and reliability of this index still require comprehensive clinical studies. The values of the thrombogenic index (TI) and atherogenic index were 1.24 and 0.47, respectively. These TI values were still ranging from 0.26 to 0.54, which are slightly higher than those of the dry “*Salchichón*” sausage containing 18% lard (TI 0.83) and the pork sausage using 100% backfat pork (TI: 1.19), as reported by Vargas-Ramella *et al*. [48].

### Microbiological characteristics

Meat is an excellent medium for the growth of microorganisms due to its rich nutritional content. Various microorganisms, such as pathogenic, spoilage, and beneficial bacteria, can be found in meat and processed products [71]. The spoilage of meat products is mainly due to microbial growth and chemical defects, such as lipid and protein oxidation. It results in sensory alterations with changes in color or texture and the development of off-odors and slime. A critical concern is the detection of high levels of pathogenic bacteria in the final product population, as certain strains of bacteria can produce toxins. The presence of these microorganisms will reduce the quality and make the final product unsafe for consumption, which can affect the level of consumer acceptance [72]. The microbiological characteristics (total aerobic mesophilic microflora, total *E. coli*, total *S. aureus*, and *Salmonella*) of *Sui Wu’u* are illustrated in Table-4.

**Table-4:** Microbiological characteristics of Sui *Wu'u* after 6 months of storage.

Total microbes (CFU/g)			

Plate count	*Escherichia coli*	*Salmonella*	*Staphylococcus aureus*
4.4 × 10^5^	<10	<10	<10

CFU=Colony-forming unit

The Indonesian Drugs and Food Supervisory Agency (BPOM: Badan Pengawas Obat dan Makanan [73] has specified maximum limits for total *E. coli*, *S. aureus*, and *Salmonella* microbes in meat and processed meat products that are not subjected to heat or fermentation processes. In contrast, there are no limits for total microbes (aerobic mesophilic flora). However, the maximum contamination limit for meat products processed by heating and freezing was 1 ´ 10^6^ colonies/g. The total aerobic mesophilic microflora of *Sui Wu’u* was 4.4 × 10^5^, which was still below the required value and lower than that of the white lawar sample of pork sold in Bali (6.7 ´ 10^5^–7.6 × 10^6^ colonies/g) [74], and traditional Spanish pork sausage [75].

*Escherichia coli* is a sanitation indicator bacteria, and its high presence in foodstuffs indicates that the food ingredients and equipment used may have been contaminated by human or animal feces. The maximum limit for *E. coli* contamination in meat and processed meat products without heating is required to be 10^3^ colonies/g [73]. Total *E.coli* in *Sui Wu’u* was undetectable (<10 CFU/g), which met the maximum limit. The presence of *E. coli* in foodstuffs has to be aware due to the severe health impact and death in humans caused by some strain, such as Shiga toxin-producing *E. coli* (STEC). However, not all STEC serotypes are pathogenic; only a small number in the entire STEC family are pathogenic. Shiga toxin-producing *E. coli* can cause diarrhea, hemorrhagic colitis, and hemolytic uremic syndrome in humans [76]. Most human infections are associated with consuming STEC-contaminated foodstuffs of animal origin. Haque *et al*. [77] stated that the global prevalence of STEC in retail pork was as high as 80%. The previous research reported that pork is a source of *E. coli* that produces the Shiga toxin [78]. Another study reported that the total *E. coli* in traditional smoked meat (beef Se’i and pork Se’i) exceeded the maximum limit required [79].

*Staphylococcus aureus* is a pathogenic bacterium that indicates food processing hygiene. The maximum limit for *S. aureus* contamination in meat and processed meat products without heating is required to be 10^4^ colonies/g [73]. Total *S. aureus* in the *Sui Wu’u* sample was undetectable (<10 CFU/g), which was still below the required maximum contamination limit. *Staphylococcus aureus* is a commensal and opportunistic pathogen that can grow over a wide range of temperatures, pH, and sodium chloride concentrations up to 15%. The amount of *S. aureus* needed to form an enterotoxin in food was about 10^6^ CFU/g. Raw pork and processed pork products are the main food sources associated with food poisoning caused by *S. aureus* [80]. *Staphylococcus aureus* causes food poisoning ranging from mild gastroenteritis to severe and potentially fatal invasive disease [81].

*Sui Wu’u’* had undetectable levels of *Salmonella* (<10 CFU/g), which conformed with BPOM regulations [72] stating that meat and processed products are required to test negative for *Salmonella* per 25 g. *Salmonella* is one of 31 pathogens that cause food poisoning (Salmonellosis). Salmonellosis is usually characterized by self-limiting gastrointestinal symptoms. Although uncommon, more severe *Salmonella* infections due to bacteremia and/or other extraintestinal infections [82] can occur and affect certain high-risk groups (infants, young children, the elderly, or patients with weak immune systems). Pork has been reported to be a source of *Salmonella* transmission to humans in various countries [83, 84].

*Sui Wu’u* is a pork product stored for a relatively long time (6 months). The number and types of microbes in *Sui Wu’u* will change after storage [85]. Microbes that survive in the final product may adapt to the product’s internal and external/environmental conditions such as temperature, a_w_, pH, salt content, and competition with other microbes [86, 87]. Various microorganisms in *Sui Wu’u* could be due to raw materials (pork and corn flour), equipment used, humans as processors, and bamboo as storage containers. The number and types of microbes in the raw materials largely determine the number and types of microbes in the final product. During processing and storage, contamination can occur through various sources such as humans, equipment, and environmental conditions. Studies such as those conducted by Bhutia *et al*. [88], and Dealino and Bueno [89] on pork sausages in the Philippines have reported the diversity of microorganisms present in traditional meat products, that is, *E. coli* (26.77%), *Salmonella* (0.33%), and *S. aureus*.

### Sensory evaluation

**Figure-1:**
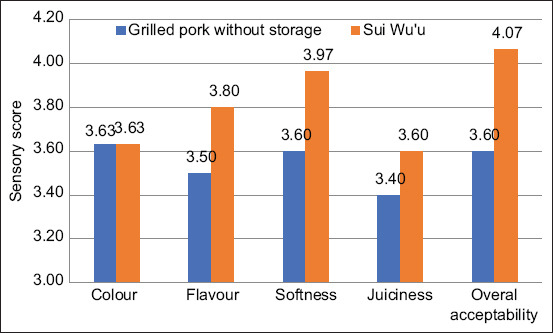
Sensory evaluation of *Sui Wu’u* after 6 months of storage.

Interestingly, panelist assessment of *Sui Wu’u* products with respect to four sensory parameters falls within the range of 3.60–4.70 (like–very like; Figure-1). This rating is higher than the sensory value of freshly grilled pork without storage (3.40–3.63).

The relatively high rating given by the panelists to the *Sui Wu’u* product, which had been stored for 6 months, was believed to be due to beneficial changes that occurred during storage. Some of these changes can be caused by the activity of microorganisms [86, 90], which, in turn, impacts the distinctive taste of *Sui Wu’u* products. *Sui Wu’u* had a relatively high-fat content (Table-1), made of pork back fat. Oxidative fat degradation will lead to the formation of several fatty acids, identified as potential pork flavor precursors, such as C 18:1n9 [91], which is the highest fatty acid found in *Sui Wu’u*. According to Pires *et al*. [92], reducing the fat content of bologna sausage by replacing 50% of the fat with *Echium oil* reduced the panelists’ acceptance of all sensory attributes. Da Silva *et al*. [93] reported similar findings by substituting pork backfat with oleogel, which is rich in oleic acid.

Salt is also a flavor enhancer that can affect the panelists’ acceptance of the final product. *Sui Wu’u*, which contains 1.7% salt content, has a preferred taste. Wang *et al*. [94] explained that low-salt fermentation improved the flavor and quality of sour meat. Low salt content (3%–5%) will affect the population and dynamics of microorganisms, thereby increasing the oxidative decomposition of proteins and lipids and facilitating the accumulation of free amino acids, organic acids, and the production of various volatile flavoring substances. The effect of salt on the salty taste of products related to the fat, protein, and water content has been very well discussed in a review by Rios-Mera *et al*. [95]. The combination of storage temperature and bamboo packaging is also believed to contribute to the panelists’ high acceptance of *Sui Wu’u*, which plays a crucial role in preventing pork from spoiling. Packaging is important for protecting the product during storage [96]. Bamboo has been used as an effective storage container in various food production processes, such as curd production from North Sumatra [97].

## Conclusion

The knowledge of curing pork for preparing the traditional food *Sui Wu’u* has been passed down from generation to generation. After 6 months of storage, *Sui Wu’u* remains a rich source of nutrients, with fat being the highest component. The highest composition of fatty acids in *Sui Wu’u* was oleic acid (C18:1n9), palmitic acid (C16:0), stearic acid (C18:0), and linoleate (C18:2n-6). The n-6/n-3 PUFA ratio is relatively high, attributed to the raw material of pork back fat. Other nutrients in *Sui Wu’u* were amino acids, such as leucine, phenylalanine, lysine, glycine, proline, glutamic acid, and alanine. The relatively low values of water content and water activity are due to the use of corn flour and salt in the production of *Sui Wu’u* and the storage in bamboo (*tuku*), contributing to the shelf life of *Sui Wu’u*. The microbiological characteristics of *Sui Wu’u* showed no detectable microorganisms (<10 CFU/g) for *Salmonella*, total *E. coli* and total *Staphylococcus*, and average values of 4.4 × 10^5^ CFU/g for total microbes, which were still below the maximum limit for microbial contamination based on the guidelines of the Food and Drug Supervisory Agency of the Republic of Indonesia. Sensory assessment for all sensory attributes showed high panelist preference (very like) for *Sui Wu’u* after being stored for 6 months. The physicochemical, microbiological, and sensory characteristics have shown that *Sui Wu’u*, after 6 months of storage, still provides the required nutrients for the body and is quite safe for consumption. The appropriate combination of pork, salt, corn flour, bamboo packaging (*tuku*), and storage temperature can maintain the stability of the shelf life of *Sui Wu’u*. The high-fat content in *Sui Wu’u* can be reduced by increasing the proportion of lean meat. The safety of *Sui Wu’u* for consumption can be further improved by incorporating strict sanitation measures in the manufacturing process, materials (pork, salt, and corn flour), and packaging (bamboo).

## Authors’ Contributions

YTR: Contributed to research design, prepared *Sui Wu’u* samples, controlled *Sui Wu’u* storage, analyzed the physicochemical, microbiological, and sensory properties, analyzed the data, and wrote the manuscript. HJDL: Contributed to research design, analyzed the physicochemical properties (amino acid and fatty acid contents), analyzed the data, and wrote the manuscript. MUES: Contributed to research design and wrote the manuscript. All authors have read, reviewed, and approved the final manuscript.
